# Deubiquitinase UCHL5 is elevated and associated with a poor clinical outcome in lung adenocarcinoma (LUAD)

**DOI:** 10.7150/jca.46146

**Published:** 2020-09-23

**Authors:** Jieru Zhang, Hui Xu, Xiaomei Yang, Yuanjie Zhao, Xinchun Xu, Ling Zhang, Xiaofeng Xuan, Chunping Ma, Wenxia Qian, Dawei Li

**Affiliations:** 1 Department of Respiratory & Critical Care Medicine, The Affiliated Zhangjiagang Hospital of Soochow University, 68 Jiyang West Road, Suzhou, 215600, China.; 2 Department of Thoracic Surgery, The Affiliated Zhangjiagang Hospital of Soochow University, 68 Jiyang West Road, Suzhou, 215600, China.; 3 Department of Emergency, The Affiliated Zhangjiagang Hospital of Soochow University, 68 Jiyang West Road, Suzhou, 215600, China.; 4 Department of General Surgery, The Affiliated Zhangjiagang Hospital of Soochow University, 68 Jiyang West Road, Suzhou, 215600, China.; 5 Department of Ultrasound, The Affiliated Zhangjiagang Hospital of Soochow University, 68 Jiyang West Road, Suzhou, 215600, China.; 6 Center for Translational Medicine, The Affiliated Zhangjiagang Hospital of Soochow University, 68 Jiyang West Road, Suzhou, 215600, China.

**Keywords:** Deubiquitinase, UCHL5/UCH37, Lung adenocarcinoma, Prognosis, Cell cycle proteins

## Abstract

Lung cancer is one of the most common malignant tumors in the world, with a high rate of malignancy and mortality. Seeking new biomarkers and potential drug targets is urgent for effective treatment of the disease. Deubiquitinase UCHL5/UCH37, as an important component of the 26S proteasome, plays critical roles in ubiquitinated substrate degradation. Although previous studies have shown that UCHL5 promotes tumorigenesis, its role in lung cancer remains largely unknown. In this study, we evaluated the expression and clinical significance of UCHL5 in non-small cell lung cancer (NSCLC). The results demonstrated that the UCHL5 expression level was significantly upregulated in NSCLC tissues compared with the adjacent noncancerous tissues. The level of UCHL5 was associated with tumor size, lymph node invasion, TNM stage and malignant tumor history in patients with lung adenocarcinoma (LUAD). Importantly, high UCHL5 expression predicted a poor overall survival (OS) and a poor disease-free survival (DFS) in patients with LUAD. Univariate regression analysis showed that tumor size, lymph node invasion, TNM stage and UCHL5 expression were associated with OS and DFS in patients with LUAD. The multivariate analysis indicated that the UCHL5 expression level was an independent prognostic factor for OS (HR=1.171, 95% CI=1.052-1.303) and DFS (HR=1.143, 95% CI=1.031-1.267) in these patients. UCHL5 knockdown in LUAD cells significantly inhibited cell proliferation and reduced the expression of key cell cycle proteins. These findings indicate that UCHL5 may serve as a potential prognostic marker and a new therapeutic target for patients with LUAD.

## Introduction

Lung cancer has become the leading cause of death from malignant tumors in the world. Non-small cell lung cancer (NSCLC) accounts for approximately 80% of all lung cancers. These include adenocarcinoma, squamous cell carcinoma and large cell carcinoma. Malignant proliferation of lung epithelial cells is a common feature of NSCLC, but the specific pathogenesis has not been fully elucidated 1. Current treatment for NSCLC includes surgery, chemoradiotherapy, immunotherapy, gene targeted therapy, etc. Because approximately 75% of patients with NSCLC are diagnosed in middle and advanced stages, the efficacy of these treatments is not good and the 5-year survival rate is very low. Therefore, it is necessary to deeply understand the pathogenesis of NSCLC and identify additional biomarkers and drug targets to improve the survival rate and prognosis of patients.

Ubiquitin C-terminal hydrolase L5 (UCHL5/UCH37) is a deubiquitination enzyme, belonging to the cysteine protease family, that exists in both the cytoplasm and nucleus. UCHL5 is an important component of the 26S proteasome, where it serves to remove distal ubiquitin moieties from ubiquitinated substrates and promote protein degradation in the 26S proteasome 2. UCHL5 is bidirectional in structure and can be activated by binding to hRPN13 while being inhibited by the Ino80 pigment remodeling complex 3-6. UCHL5 is functionally linked with multiple protein complexes and signaling transduction pathways. UCHL5 is involved in the activation of the Wnt, Hedgehog and transforming growth factor-β (TGF-β) signaling pathways. UCHL5 regulates gene transcription and DNA damage repair and replication 7-11.

Accumulating evidence indicates that UCHL5 plays an important role in tumorigenesis, tumor invasion and migration 12-16. A high UCHL5 expression level predicts early recurrence and promotes cell migration and invasion in hepatocellular carcinoma (HCC) 12. UCHL5 can be a predictor of poor survival in esophageal squamous cell carcinoma (ESCC) after curative resection 13. UCHL5 is a potential marker of gastric cancer and is also involved in the occurrence and development of colon cancer, cervical cancer and multiple myeloma 14-16. A small molecule inhibitor targeting UCHL5 and USP14 induced cell apoptosis and ameliorated the disease progression of multiple myeloma 16.

In this study, we aimed to investigate the expression of UCHL5 in NSCLC tissues and its correlation with the clinicopathological characteristics of patients with NSCLC. Furthermore, we investigated the molecular events by which UCHL5 is implicated in controlling the growth of LUAD cells. We propose that UCHL5 may serve as a new biomarker to evaluate the progression of the disease and as a potential drug target for treating LUAD.

## Materials and methods

### Patients

We evaluated UCHL5 expression in NSCLC tissues and the adjacent paired non-cancerous tissues from 25 patients. Patients who underwent curative surgery at The Affiliated Zhangjiagang Hospital of Soochow University from March 2019 to February 2020 and were clearly diagnosed with NSCLC by pathology were included. Each sample was reviewed by at least a pathologist to confirm the diagnosis. None of the above patients received adjuvant chemotherapy before surgery. Their tumor staging was assessed at diagnosis according to the American Joint Commission on Cancer Guidelines (http://www.cancerstaging.org/). The current study focuses on primary untreated tumors that were snap frozen upon collection. All tumors must have a matched normal sample from the same patient. Tissue samples included the noncancerous lung tissues taken far from the primary tumor site of NSCLC tissues. Tissue samples were immediately stored at -80°C for mRNA and protein extraction and immunostaining. The use of the human specimens was approved by The Zhangjiagang Hospital Institutional Review Board (No. 2019001). All of the patients signed the informed consent form. The demographic and clinical features of the patients are shown in Supplementary Table 1.

### Bioinformatics analysis

We also made use of the publicly available clinical information provided by the cBioportal for Cancer Genomics (TCGA) database (http://www.cbioportal.org/) 17, 18 , which includes basic demographic and clinical information, as well as survival status after surgery, to assess the correlation between the UCHL5 expression level and clinical pathological status as well as the patients' overall survival in NSCLC. We retrieved clinical information from non-small cell lung carcinoma (NSCLC) patients including 510 patients with lung adenocarcinoma (LUAD) and 501 patients with lung squamous cell carcinoma (LUSC) for correlation analysis. The demographic and clinical features of the patients and UCHL5 expression are shown in Supplementary Table 2 and 3. We stratified 494 and 425 patients with LUAD into higher and lower UCHL5 expression levels for the overall survival (OS) and disease-free survival (DFS) analyses, respectively. We also stratified 495 and 375 patients with lung squamous cell carcinoma (LUSC), respectively, for the survival analysis. Finally, a total of 460 and 391 patients with LUAD with complete demographic and clinical information were used for univariate and multivariate Cox regression analysis for OS and DFS, respectively.

### Cell culture

Human NSCLC cell lines H1299 and A549 were cultured in Dulbecco's modified Eagle's medium (DMEM) supplemented with 10% fetal bovine serum (FBS) and antibiotics (100 U/ml penicillin and 100 µg/ml streptomycin). All cells were cultured at 37°C in a humidified incubator with 5% CO_2_.

### Real-time PCR (RT-PCR)

Total RNA was isolated from tissues or cultured cells using Trizol reagent (Invitrogen, Carlsbad, CA, USA) following the manufacturer's protocol and was reverse transcribed into cDNA using the Thermo Scientific Revertaid H Minus First Strand cDNA Synthesis Kit (Thermo Fisher, Waltham, MA, USA ). Real-time PCR (RT-PCR) was performed in triplicate using SYBR green mix (Applied Biosystems, Foster City, CA, USA) and a QuantStudio Dx Real-Time PCR Instrument (Applied Biosystems) under the following conditions: 10 min at 95°C, followed by 40 cycles of 95°C for 15 s and 60°C for 1 min. β-Actin was used as an internal reference for normalization. The sequences of the primers (Sangon, Shanghai, China) were as follows: UCHL5, F: 5'-GAACGCAAAGAAAGCTCAGG-3', R: 5'-AGACAAGACAGGCTGGCACT-3'; β-Actin, F: 5'-AGAGCTACGAGCTGCCTGAC-3', R: 5'-AGCACTGTGTTGGCGTACAG-3'; Cyclin D1, F: 5'-ACGAAGGTCTGCGCGTGTT-3', R: 5'-CCGCTGGCCATGAACTACCT-3'; CDK4, F: 5'-CCTGGCCAGAATCTACAGCTA-3', R: 5'-ACATCTCGAGGCCAGTCATC-3'; Cyclin B1, F: 5'-AAGAGCTTTAAACTTTGGTCTGGG-3', R: 5'-CTTTGTAAGTCCTTGATTTACCATG-3'; CDK1, F: 5'-TGGATCTGAAGAAATACTTGGATTCTA-3', R: 5'-CAATCCCCTGTAGGATTTGG-3'; Cdc25C, F: 5'-GATGTCCCTAGAACTCCAGTG-3', R: 5'-AGTTATCTCCCCACTGCTAAGA-3'; RB, F: 5'-AGGATCAGATGAAGCAGATGG-3', R: 5'- TGCATTCGTGTTCGAGTAGAAG-3'; p21, F: 5'-CCATGTGGACCTGTCACTGTCTT-3', R: 5'-CGGCCTCTTGGAGAAGATCAGCCG-3'; p27, F: 5'-TTTGACTTGCATGAAGAGAAGC-3', R: 5'-AGCTGTCTCTGAAAGGGACATT-3'.

### Western blotting and antibodies

The tissues and cells were collected and homogenized in FLAG lysis buffer (50 mM Tris·HCl, pH 7.9, 137 mM NaCl, 10 mM NaF, 1 mM EDTA, 1% Triton X-100, 0.2% Sarkosyl, 10% glycerol) containing a protease inhibitor cocktail (Sigma, St. Louis, Missouri, USA). The proteins were extracted and their concentration was measured using a BCA protein assay kit (Pierce, Rockford, IL, USA). Equal quantities of protein from each sample were loaded for SDS-PAGE. After electrophoresis, the proteins were transferred onto nitrocellulose membranes (GE Healthcare, Munich, Germany). The membranes were blocked in 5% nonfat milk for 1 h at room temperature (RT) and subsequently incubated overnight at 4°C with primary antibodies against: UCHL5 (sc-271002); Cyclin D1 (sc-450); Cdk4 (sc-23896); Cyclin B1 (sc-7393); CDK1 (sc-54); Cdc25C (sc-13138); RB (sc-74562); p21 (sc-53870) and p27 (sc-1641) from Santa Cruz Biotechnology (CA, USA); P-RB (Ser807/811, Cell Signaling Technology, Danvers, MA, USA); and β-Actin (A3853, Sigma-Aldrich, Darmstadt, Germany). After extensive washing, the membranes were incubated with the appropriate secondary antibodies for one hour at RT. The target band signals were developed using enhanced chemiluminescence (ECL, WBKLS0500, Millipore, Bedford, MA, USA) and a ChemiDoc XRS (Bio-Rad, CA, USA) detection system. The signals were quantified using ImageJ software (National Institutes of Health, Bethesda, MD, USA).

### Immunohistochemistry (IHC)

Frozen sections with a thickness of 6 µm were fixed in 4% formaldehyde for 15 min at room temperature (RT). The sections were washed twice for 5 min each in phosphate buffered saline (PBS) containing 0.3% Triton X-100 and then blocked with 10% normal goat serum for one hour at RT. The sections were incubated with anti-UCHL5 primary antibody at a dilution of 1:100 overnight at 4°C and further treated with 0.3% H_2_O_2_ for 15 min. After extensive washing, the biotin conjugated secondary antibody was added to the sections, which were further incubated for one hour at RT. After extensive washing, a solution containing streptavidin and biotin conjugated HRP (Beyotime, China) was added to the sections. The sections were incubated for one hour at RT and then washed in PBS 3 times for 5 min each. The slides were developed with 3'-diaminobenzidine (DAB, Beyotime, China) for 30 min at RT followed by rinsing in H_2_O for 5 min. The slides were counterstained in hematoxylin, dehydrated, cleared and mounted. Immunohistochemical staining was observed using a Leica upright microscope (DM4000B, Leica Microsystems, Heidelberg, Germany). UCHL5 IHC staining was also performed by tissue microarrays from a large cohort of 75 LUAD patients (HLugA150CS03, Shanghai Outdo Biotech Company, Shanghai, China). The demographic and clinical information for these patients was shown in Supplementary Table 4. The protein staining percentage was scored from 0 to 3 points for each section. A score of 0 was given to no positive cells or a percentage of positive cells ≤5%. A score of 1 was given to cases whose percentage of positive cells was 6%-25%. If the average percentage of positive cells was >25% but ≤50%, then the expression was scored as 2, whereas the percentage of positive cells >50% but ≤75% was scored as 3; and >75% positive staining percentage was scored as 4. Protein staining density was scored from 0 to 2: no staining was scored as 0; light brown as 1 and dark brown as 2. The final scores were calculated as the product of the staining percentage and intensity scores for each section. All of the sections were independently assessed by two pathologists. A mean score was used if controversial results were acquired.

### RNA interference

H1299 and A549 cells were transfected with specific siRNAs using Lipofectamine 2000 (Invitrogen, USA) according to the manufacturer's protocol. Two individual UCHL5 siRNAs (siUCHL5-1 and siUCHL5-2) and a scrambled negative control siRNA (NC) were synthesized by GenePharma (Shanghai, China). The nucleotide sequences of the siRNAs were as follows: siUCHL5-1: sense, 5'-GCAAAGAAAGCUCAGGAAATT-3', antisense, 5'-UUUCCUGAGCUUUCUUUGCTT-3'; siUCHL5-2: sense, 5'-GAUCAAGGUAAUAGUAUGUTT-3', antisense, 5'-ACAUACUAUUACCUUGAUCTT-3'; and NC: sense, 5'-UUCUCCGAACGUGUCACGUTT-3', antisense, 5'-ACGUGACACGUUCGGAGAATT-3'.

### Cell viability and proliferation assays

Cell viability was detected using a cell counting kit-8 (CCK-8, Dojindo, Kumamoto, Japan). H1299 and A549 cells were seeded at a density of 1000 cells per well in 96-well plates. After 24 h, the cells were transfected with siRNAs using Lipofectamine 2000 reagent following the manufacture's protocol. Cell viability was monitored at 1, 3 and 5 days after the cells were transfected with scrambled or UCHL5 siRNAs. CCK-8 optical density (OD) at 450 nm was measured with a microplate reader (Bio-Rad Laboratories, USA).

### Statistical analysis

All data are presented as the mean ± SD. GraphPad Prism 8.0 software was used for statistical analysis. The results were analyzed using Student's t test for comparison of two groups or one-way ANOVA for comparisons of multiple groups. The relationships between UCHL5 expression and clinical pathological parameters were analyzed by the Chi-square or Fisher's exact test. Survival curves were plotted using the Kaplan-Meier method and compared by the log-rank test. Univariate and multivariate Cox regression analysis were used to assess variables associated with OS and DFS of LUAD patients. Statistical significance was accepted at p< 0.05.

## Results

### The UCHL5 expression level is upregulated in human NSCLC tissues

**Figure 1:**
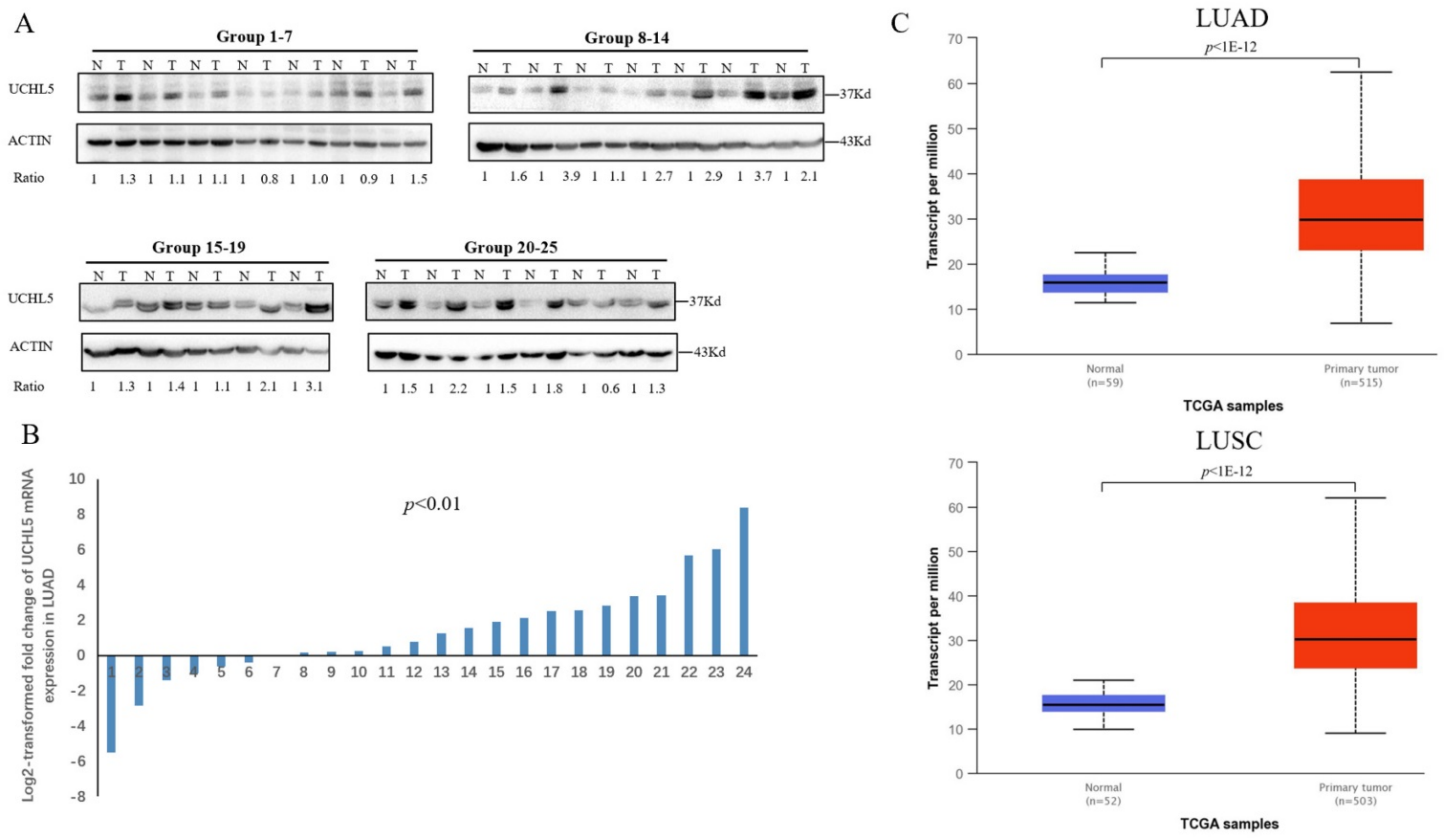

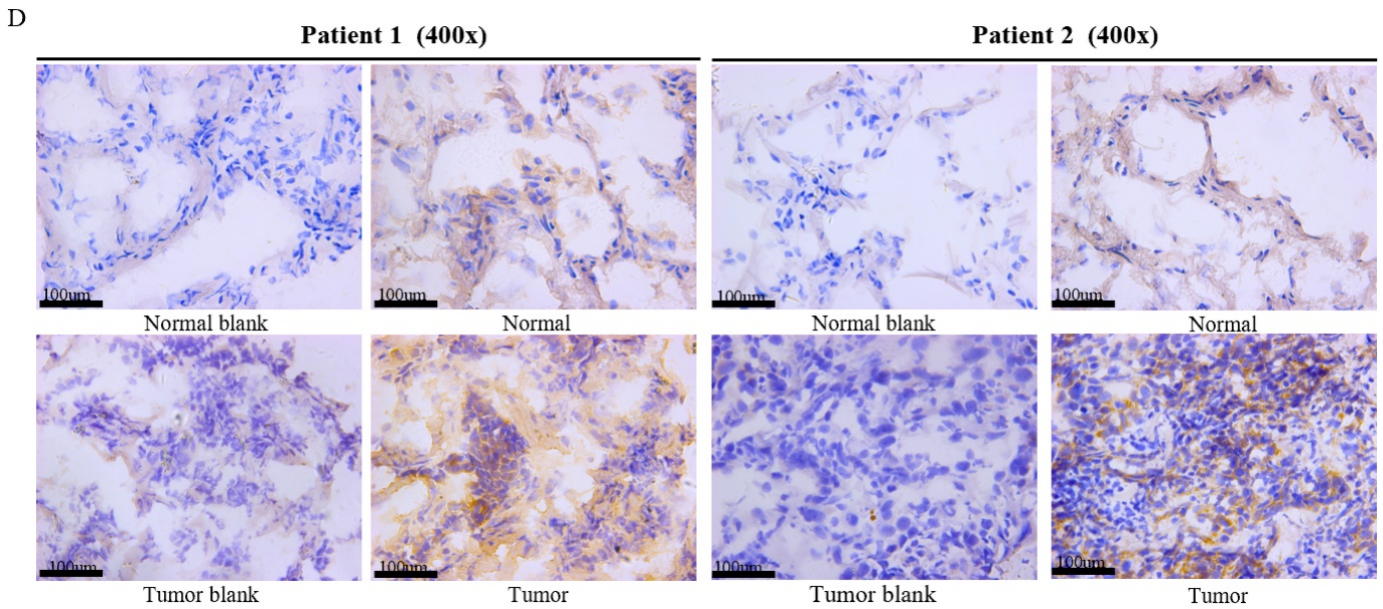
UCHL5 expression is upregulated in NSCLC tissues. (A) UCHL5 expression in 25 paired tumor and adjacent noncancerous tissues from patients with NSCLC was determined by western blotting. β-Actin was used as an internal control. (B) Relative mRNA expression of UCHL5 in 24 pairs of NSCLC tumor tissues and adjacent noncancerous control tissues was determined by RT-PCR (p<0.01 from Fisher's exact test). (C) Box (25-75th percentiles) and whisker (minimum-maximum) plots for UCHL5 expression in controls and NSCLC (LUAD and LUSC) patients; the horizontal line inside the box indicates the median (the 50th percentile). P-value calculated by Kruskal-Wallis test. (D) The typical IHC staining of UCHL5 in the paired tumor and adjacent non-cancerous tissues. The staining was performed in two pairs of tissues and a representative photograph was shown. Bar represents 100 µm. LUAD: lung adenocarcinoma; OS: overall survival; DFS: disease-free survival.

**Figure 2:**
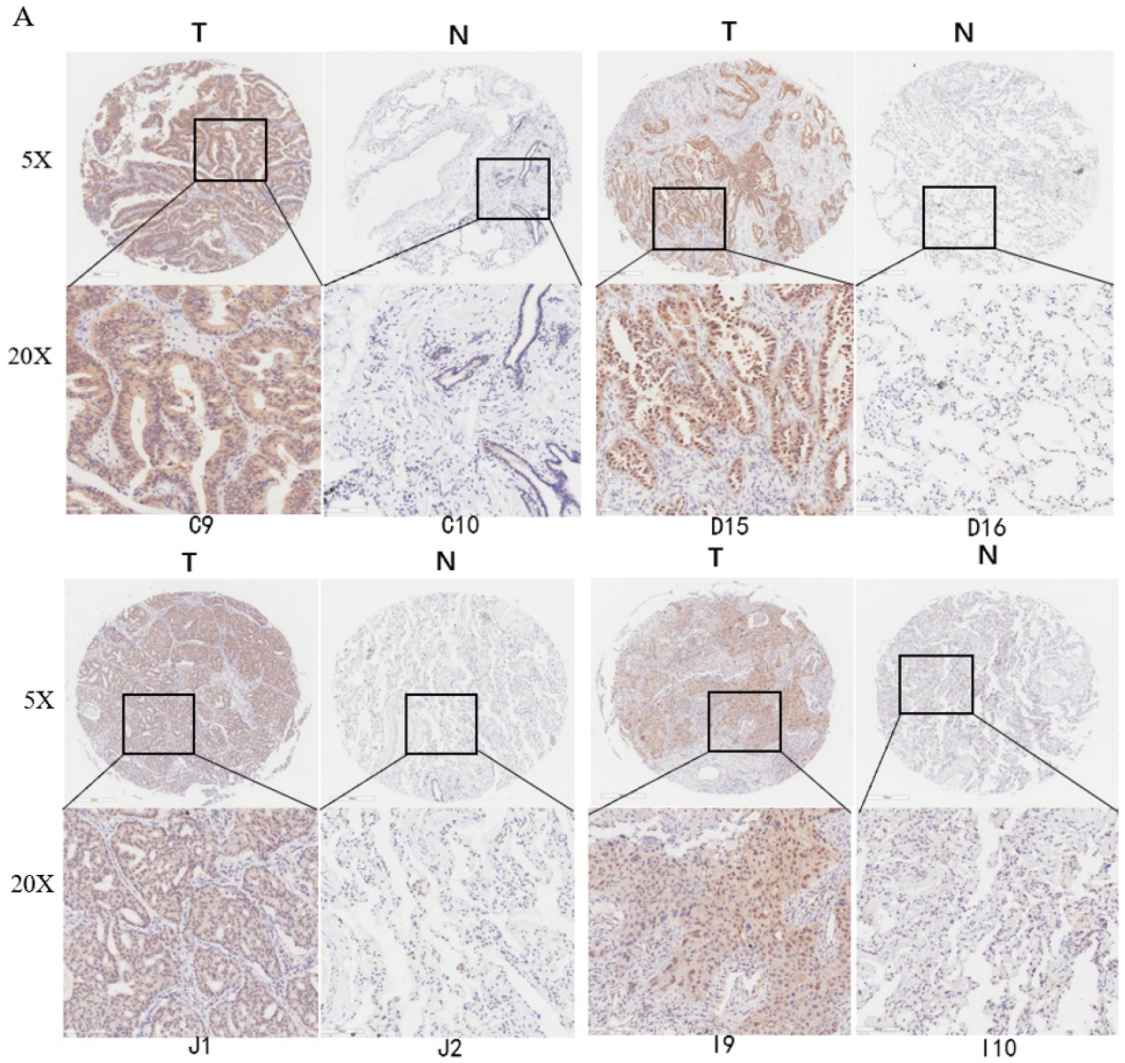

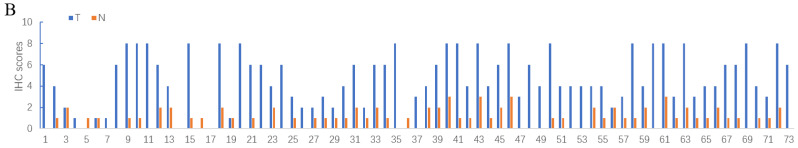
UCHL5 expression is upregulated in LUAD tissues by tissue microarray. (A) Typical IHC staining of UCHL5 in the paired tumor and adjacent non-cancerous tissues. The staining was performed in 75 pairs of tissues and representative pictures from four pairs (C9 and C10; D15 and D16; J1 and J2; I9 and I10) were shown. UCHL5 staining in 2 paired tissues (B7 and B8; F7 and F8) out of 75 paired tissues from LUAD patients was missing. The bar represents 100 µm. (B) Relative IHC scores of UCHL5 in 73 pairs of lung cancer tissues (T) and adjacent non-cancerous tissues (N) were analyzed. NSCLC: Non-small cell lung cancer.

Deubiquitinase UCHL5 is a component of the 26S proteasome, and it is involved in regulating the degradation of ubiquitinated substrates. Although previous studies have indicated that UCHL5 is implicated in tumorigenesis, invasion and metastasis 12-16, its expression in NSCLC tissues remains undetermined. We first investigated UCHL5 expression in tumor and adjacent noncancerous control tissues from 25 patients with NSCLC by RT-PCR and western blot. The results demonstrated that UCHL5 protein expression is significantly higher in tumor tissues in comparison with the control tissues from patients with NSCLC (Figure 1A). UCHL5 was upregulated in 21 tissues (84%) and downregulated in 4 tissues (16%) out of 25 tumor tissues (Figure 1A, p<0.001 by Fisher's exact test). Consistently, the UCHL5 mRNA level was markedly elevated in 18 (75%) and decreased in 6 (25%) out of 24 tumor tissues in comparison with the controls (Figure 1B, p<0.01 by Fisher's exact test). We compared UCHL5 mRNA expression in normal and tumor tissues of NSCLC patients using TCGA database. 59 normal lung tissues and 519 tumor tissues from LUAD patients and 52 normal lung tissues and 503 tumor tissues from LUSC patients were analyzed. UCHL5 level was significantly elevated in tumor tissues from LUAD and LUSC patients compared with normal lung tissues (Figure 1C). We also performed UCHL5 IHC staining on tumor tissues and paired non-cancerous control tissues (controls) of NSCLC patients. UCHL5 staining in tumor tissues was much stronger than that in controls (Figure 1D). Higher UCHL5 expression was mainly detected in cytoplasm of tumor cells. To further support the conclusion, we conducted an IHC analysis using tissue microarrays from a large cohort of 75 LUAD patients. The UCHL5 IHC staining from 2 patients was missing from the microarray. UCHL5 expression was detected in both tumor and paired non-cancerous tissues from 73 LUAD patients (Figure 2A and Supplementary Figure 1). The results demonstrated that UCHL5 expression was upregulated in 64 (87.7%), unchanged in 6 (8.2%) and downregulated in 3 (4.1%) tumor tissues in comparison with the paired non-cancerous tissues out of 73 patients (p<0.0001, chi-squared test; Figure 2B). Thus, the UCHL5 level was significantly elevated in NSCLC tumor tissues in comparison with non-cancerous tissues.

### UCHL5 expression is associated with the clinical features of patients with LUAD

Next, we made use of the TCGA database to evaluate the relationship between UCHL5 expression and the demographic and clinical characteristics of patients with NSCLC. The results showed that the UCHL5 expression level was associated with tumor size, lymph node metastasis, TNM stage and malignant tumor history in patients with LUAD (Table 1). However, there was no correlation between UCHL5 expression and the patients' demographic and clinicopathological characteristics in LUSC (Table 1). Thus, higher UCHL5 expression reflects severe and progressive disease in patients with LUAD.

### High UCHL5 expression indicates a poor prognosis of patients with LUAD

**Figure 3:**
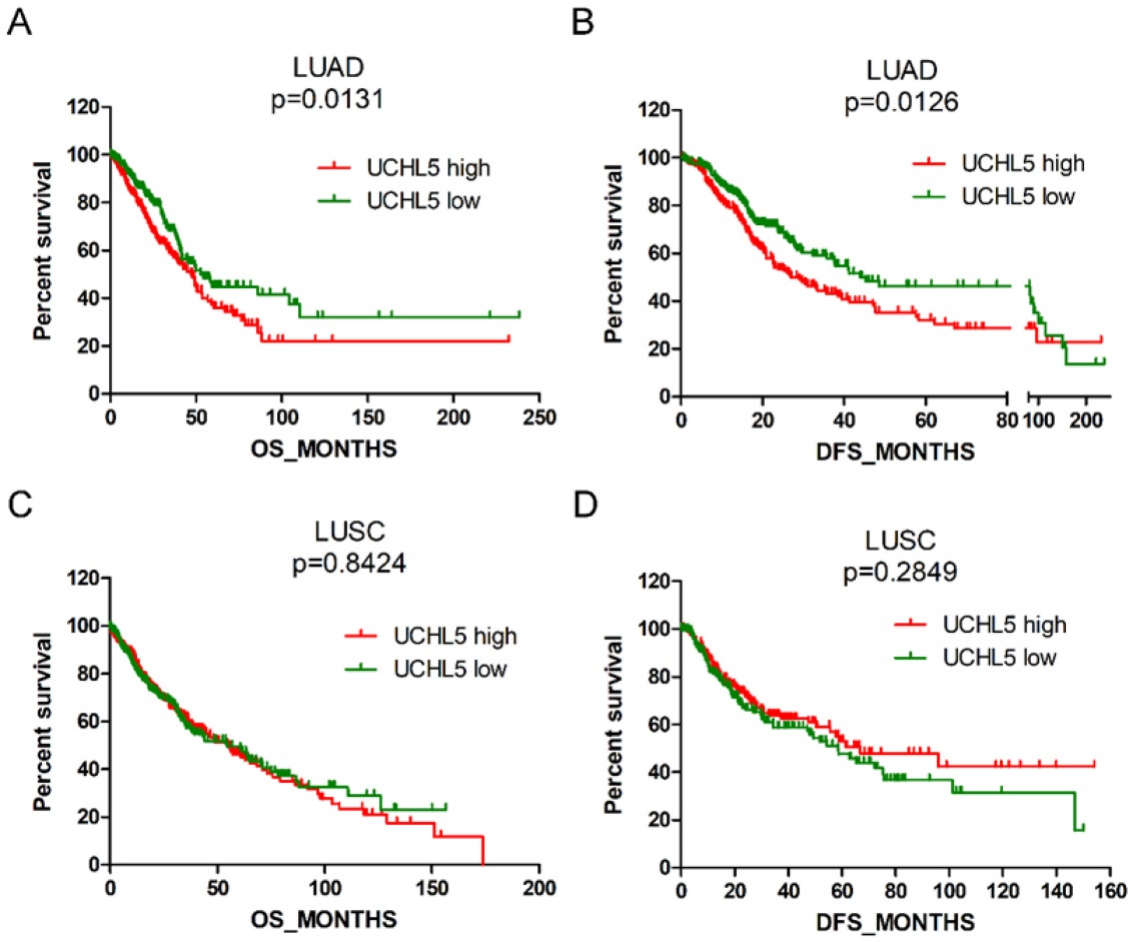
High UCHL5 expression indicates a poor OS and DFS in patients with LUAD. (A-B) Kaplan-Meier survival curves of higher and lower UCHL5 expression for OS and DFS in patients with LUAD. (C-D) Kaplan-Meier survival curves of higher and lower UCHL5 expression for OS and DFS in patients with LUSC. The patients' information was retrieved from the cBioportal for the Cancer Genomics (TCGA) database and analyzed by a log-rank test.

We evaluated the prognostic significance of UCHL5 expression in patients with NSCLC using data from the TCGA database. The results indicated that the patients with LUAD with higher levels of UCHL5 expression had a worse OS and DFS than those with a lower UCHL5 level (Figure 3A and B). The median OS and DFS were 47.8 and 26.9 months for patients with higher UCHL5 levels and 54.3 and 44.0 months for patients with lower UCHL5 levels, respectively. However, there was no significant correlation between the level of UCHL5 expression and OS and DFS of patients with LUSC (Figure 3C and D). Univariate Cox regression analysis indicated that tumor size, lymph node invasion, TNM stage and UCHL5 level were correlated with OS and DFS in patients with LUAD. Multivariate Cox regression analysis including the above parameters showed that lymph node invasion, TNM stage and UCHL5 level were independent prognostic factors for OS and tobacco smoking, and tumor size and UCHL5 expression were independent prognostic factors for DFS in these patients. The UCHL5 level was an independent prognostic factor for OS (HR=1.171, 95% CI=1.052-1.303) and DFS (HR=1.143, 95% CI=1.031-1.267) of patients with LUAD.

### UCHL5 knockdown inhibits LUAD cell proliferation via regulation of cell cycle proteins

**Figure 4:**
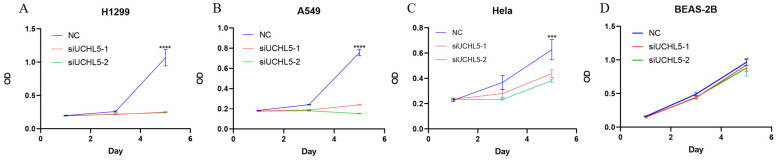

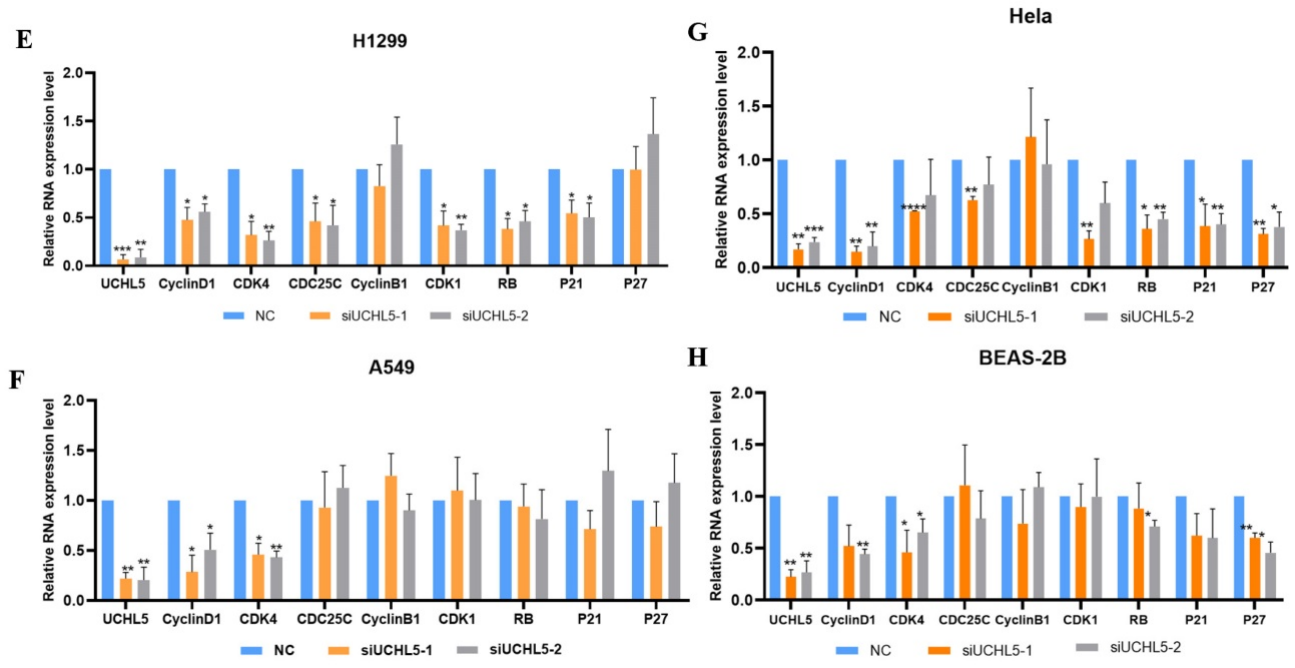

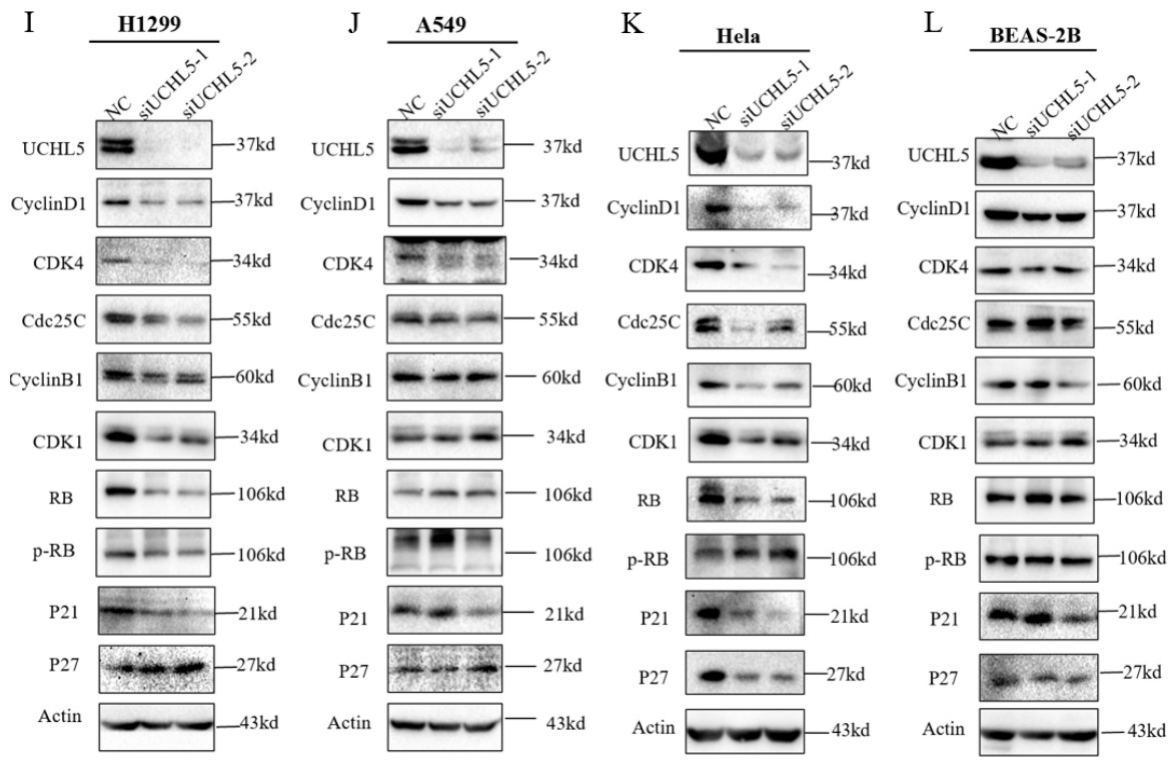
UCHL5 knockdown inhibits cell growth by regulating cell cycle proteins in LUAD cells. (A-D) The cell viability of H1299, A549, Hela and BEAS-2B cells transfected with NC, si-UCHL5-1 and si-UCHL5-2 was assessed by the CCK-8 assay. The OD value was determined at 1 day, 3 days and 5 days post transfection. The assays were performed in four replicates for each group. The experiments were repeated twice, and a representative result is shown. (E-H) The H1299, A549, Hela and BEAS-2B cells were transfected with NC, si-UCHL5-1 and si-UCHL5-2 for 72 h, and the mRNA expression levels of related molecules were detected by RT-PCR. (I-L) The whole-cell lysates from the H1299, A549, Hela and BEAS-2B cells were extracted after the cells were transfected with NC, si-UCHL5-1 and si-UCHL5-2 for 72 h. Western blot analysis was performed with the indicated antibodies. Actin was used as an internal control. NS: not significant; NC: scrambled control siRNA. *, p<0.05; **, p<0.01; ***, p<0.001 and ****, p<0.0001 by Student's t test.

Finally, we chose two LUAD cell lines, H1299 and A549, to investigate the role of UCHL5 in the pathogenesis of LUAD. The results showed that cell proliferation was significantly inhibited after UCHL5 expression was knocked down by two different siRNA fragments in the H1299 and A549 cell lines (Figure 4A and B). To clarify the molecular events by which UCHL5 regulates cell growth, we detected the key cell cycle proteins in both cell lines. The results demonstrated that the protein levels of cell cycle-related regulators including Cyclin D1, CDK4, Cyclin B1, CDK1, Cdc25C, p21, RB and phosphorylated RB (p-RB) were significantly decreased, whereas the p27 level increased after UCHL5 was knocked down in H1299 cells (Figure 4I). However, only Cyclin D1 and CDK4 protein expression were inhibited after UCHL5 was knocked down in A549 cells (Figure 4J). Consistently, we observed that the mRNA levels of Cyclin D1, CDK4, CDK1 and Cdc25C were decreased whereas Cyclin B1 and p27 levels remained the same after UCHL5 knockdown in H1299 cells (Figure 4E). The mRNA expression levels of Cyclin D1 and CDK4 expression were reduced after UCHL5 was knocked down in A549 cells (Figure 4F). These results suggest that the UCHL5-mediated regulation of cell cycle proteins is cell-type dependent. Finally, we assessed the UCHL5 effects on cell growth in the other tumor type and BEAS-2B lung epithelial cells. UCHL5 knockdown by two siRNA segments significantly inhibited cell proliferation in Hela cells (Figure 4C). Consistently, the mRNA and protein level of Cyclin D1, CDK4, Cdc25C, p21, p27 and RB were markedly inhibited after UCHL5 was depleted from Hela cells (Figure 4G and 4K). However, we did not observe significant growth inhibiting effects after UCHL5 was knocked down from BEAS-2B lung epithelial cells (Figure 4D). The mRNA and protein level of Cyclin D1 and CDK4 was significantly inhibited in UCHL5 knocked down BEAS-2B cells (Figure 4H and L).

## Discussion

Lung cancer is one of the most common malignant tumors in the world. The vast majority of patients have non-small cell lung cancer (NSCLC), for which growth is relatively slow and metastasis occurs late. However, because the early symptoms are not obvious, these cases are mostly found in the middle and late stages, and the patient prognosis is poor. Therefore, it is urgent to find specific molecular markers to assess the progression of the disease. UCHL5 is a deubiquitination enzyme in the 26S proteasome, which directly affects the regulation and renewal of proteins. In this study, we found that UCHL5 was significantly upregulated in NSCLC tissues. A high expression level of UCHL5 was closely associated with a larger tumor size, early lymph node metastasis and advanced TNM, which predicted poor OS and DFS of patients with LUAD. All of the above results suggested that UCHL5 may have a promoting effect on NSCLC and may be a potential prognostic marker.

The deubiquitinases UCHL5, PSMD14, PSMD7 and USP14 are located in the 19S regulatory particles of the 26S proteasome, which modulates ubiquitinated substrate degradation 19. Our previous study revealed that PSMD14 is upregulated and may serve as a prognostic marker for LAUD 20. In this study, we found that UCHL5 is elevated and has prognostic significance in LUAD. In combination, these findings suggest that the key components of the 26S proteasome may be involved in tumorigenesis and progression in patients with LUAD. Thus, small molecule inhibitors against components of the 26S proteasome may be potential drug targets for effective treatment of LUAD 21-22. In support of this suggestion, several components of the 26S proteasome have been identified as therapeutic targets for malignant tumors. An example is that bortezomib, a reversible inhibitor of the chymotrypsin-like activity of PSMD5 in the proteasome, has been widely used in the treatment of multiple myeloma 23-24. Recently, novel small molecule inhibitors of the deubiquitylating enzymes USP14 and UCHL5 were developed to overcome bortezomib resistance and induce cell apoptosis of multiple myeloma 16, 25. Our findings suggest that UCHL5 may be a promising target for therapeutic intervention in LUAD.

Cyclins are a group of proteins that control the progression of cells through the cell cycle by activating cyclin-dependent kinase (CDK) enzymes. Cyclin D/CDK4 regulates the transition from G1 to S phase. RB, a G1 phase cycle inhibitor, is phosphorylated and activated, which disables the inhibition of transcription factors, and they begin to transcribe cell cycle genes, thus promoting the cell from G1 phase to S phase. Cyclin B/CDK1 regulates the progression from G2 to M phase 26-28. P21 and p27 are members of the CDK inhibitor (CKI) and negatively regulate the cell cycle 29-30. In addition, CDC25 (cell division cycle 25) is also involved in cell cycle regulation, making the cells stay in a particular phase 31. Degradation of all of the above cyclins occurs through the ubiquitin-proteasome pathway 26. To further understand the role of UCHL5 in NSCLC, we investigated the effects of UCHL5 knockdown on cell cycle proteins in LUAD cells. We found that the expression of cell cycle-related proteins including Cyclin D1, CDK4, Cyclin B1, CDK1, Cdc25C, RB and p21 was markedly inhibited, thus leading to inhibition of cell proliferation in H1299 cells. However, UCHL5 knockdown only blocked the expression of Cyclin D1 and CDK4 in A549 cells, suggesting that the effects of UCHL5 on cell cycle proteins is cell-type specific. Importantly, we observed cell growth is markedly inhibited in UCHL5 knocked down tumor cells but not in lung epithelial cell BEAS-2B, highlighting usefulness of UCHL5 as a potential therapeutic target for tumors. Although cyclin D1 and CDK4 were significantly inhibited, we did not observe difference in cell growth after UCHL5 was knocked down from BEAS-2B cells. Previous studies indicate that tumor cells are addicted to cyclin D-CDK kinase for cell survival 32-33. Consistently, we observed decreased cyclin D1 and CDK4 levels and delayed cell growth in UCHL5 knocked down tumor cells. However, we did not observe significant growth inhibition effects in lung epithelial cells although both proteins were inhibited, suggesting that cyclin D1-CDK4 kinase activity is not absolutely required for the growth of those cells.

Although UCHL5 is a deubiquitinase in the 26S proteasome involved in protein degradation, we did not observe significant stabilization of cell cycle proteins, except for p27, after UCHL5 was knocked down in LUAD cells. In contrast, we found that the transcription level of the related genes was significantly inhibited after UCHL5 was knocked down. The underlying molecular events by which UCHL5 regulates gene transcription remain to be further investigated.

## Conclusions

In conclusion, our study showed that UCHL5 expression is elevated in NSCLC and predicts a poor prognosis of patients with LUAD. UCHL5 knockdown markedly inhibited cell proliferation via regulating cell cycle proteins. Therefore, UCHL5 is expected to become a potential prognostic marker and a new therapeutic target for LUAD.

## Supplementary Material

Supplementary figure.

Supplementary table 1.

Supplementary table 2.

Supplementary table 3.

Supplementary table 4.

## Figures and Tables

**Table 1:** The expression of UCHL5 was correlated with demographic and clinical pathological characteristics in patients with NSCLC (LUAD and LUSC)

	LUAD UCHL5				LUSC UCHL5		
	high	low	p value		high	low	p value
**Age**							
≥60	175	180	0.666		196	205	0.296
<60	70	66			50	41	
**Sex**							
Male	129	107	0.051		192	179	0.212
Female	126	148			59	71	
**Tabaco smoking history**							
Stage 3-5	161	143	0.097		166	172	0.513
Stage 1-2	87	105			79	72	
**Other malignancy history**							
Negative	242	228	0.031*		218	215	0.694
Positive	13	27			32	35	
**Laterality**							
Left	92	107	0.120		114	99	0.165
Right	155	141			122	137	
**Location of lung parenchyma**							
Peripheral lung	60	65	0.440		47	46	0.895
Central lung	34	29			73	74	
**Residual tumors**							
Negative (R0)	170	171	0.991		199	199	1.000
Positive (R1/R2)	8	8			8	8	
**Tumor size**							
T1	66	101	0.001*		50	64	0.142
T2-T4	187	153			200	187	
**Lymph Node Stage**							
Negative	151	177	0.017*		155	164	0.433
Positive	98	73			92	84	
**Distant metastasis**							
Negative	167	174	0.147		204	207	0.253
Positive	16	9			5	2	
**Tumor Stage**							
Ⅰ/Ⅱ	188	205	0.040*		201	205	0.617
III/IV	63	46			48	43	

The clinical information from the patients with NSCLC (510 patients with LUAD and 501 with LUSC) was retrieved from the cBioportal for Cancer Genomics (TCGA) databases for correlation analysis. NSCLC: non-small cell lung carcinoma; LUAD: lung adenocarcinoma; LUSC: lung squamous cell carcinoma. *p<0.05 was considered significant.

**Table 2:** Univariate and multivariate Cox regression analysis of prognostic factors of survival of patients with LUAD

Parameters	Overall survival (OS, n=460)			Disease-free survival (DFS, n=391)	
	HR (95% CI)	p value		HR (95% CI)	p value
**Univariate regression analysis**					
Age	1.280 (0.887-1.848)	0.188		1.441 (1.020-2.037)	0.038^*^
Sex	1.134 (0.814-1.578)	0.457		0.993 (0.727-1.356)	0.963
Tobacco smoking	1.176 (0.834-1.658)	0.355		1.347 (0.972-1.867)	0.073
Other malignancy	1.314 (0.835-2.066)	0.238		1.220 (0.814-1.829)	0.335
Tumor size	1.639 (1.122-2.394)	0.011^*^		1.932 (1.359-2.746)	<0.001^*^
Lymph node invasion	2.534 (1.816-3.534)	<0.001^*^		1.651 (1.199-2.273)	0.002^*^
TNM stage	1.579 (1.347-1.850)	<0.001^*^		1.325 (1.125-1.561)	0.001^*^
UCHL5 expression	1.289 (1.086-1.347)	0.001^*^		1.161 (1.050-1.283)	0.004^*^
**Multivariate regression analysis**					
Age	-	-		1.385 (0.974-1.969)	0.070
Tobacco smoking	-	-		1.410 (1.011-1.966)	0.043^*^
Tumor size	1.304 (0.886-1.921)	0.179		1.700 (1.176-2.458)	0.005^*^
Lymph node invasion	1.795 (1.178-2.735)	0.007^*^		1.310 (0.862-1.933)	0.206
TNM stage	1.260 (1.016-1.563)	0.036^*^		1.119 (0.892-1.403)	0.331
UCHL5 expression	1.171 (1.052-1.303)	0.004^*^		1.143 (1.031-1.267)	0.011^*^

Patients with LUAD with complete demographic and clinical information were extracted from the cBioportal for Cancer Genomics (TCGA) database for univariate and multivariate Cox regression analysis. HR: hazard ratio, CI: confidence interval. *p<0.05 was considered significant.

## Abbreviations

UCHL5/UCH37: ubiquitin C-terminal hydrolase L5
NSCLC: non-small cell lung cancer
LUAD: lung adenocarcinoma
LUSC: lung squamous cell carcinoma
OS: overall survival
DFS: disease-free survival
GEO: Gene Expression Omnibus
TCGA: The Cancer Genome Atlas
DMEM: Dulbecco's modified Eagle's medium
FBS: fetal bovine serum
RT-PCR: real-time PCR
ECL: enhanced chemiluminescence
IHC: immunohistochemistry
PBS: phosphate buffered saline
