# Supplementary material for: Deubiquitinase UCHL5 is elevated and associated with a poor clinical outcome in lung adenocarcinoma (LUAD)
Source: J Cancer. 2020 Sep 23;11(22):6675–85. doi: 10.7150/jca.46146 (PMC7545677; doi:10.7150/jca.46146)
Supplement: Supplementary file 1 — Supplementary figure. [file jcav11p6675s1.pdf]

Supplementary Figure 1

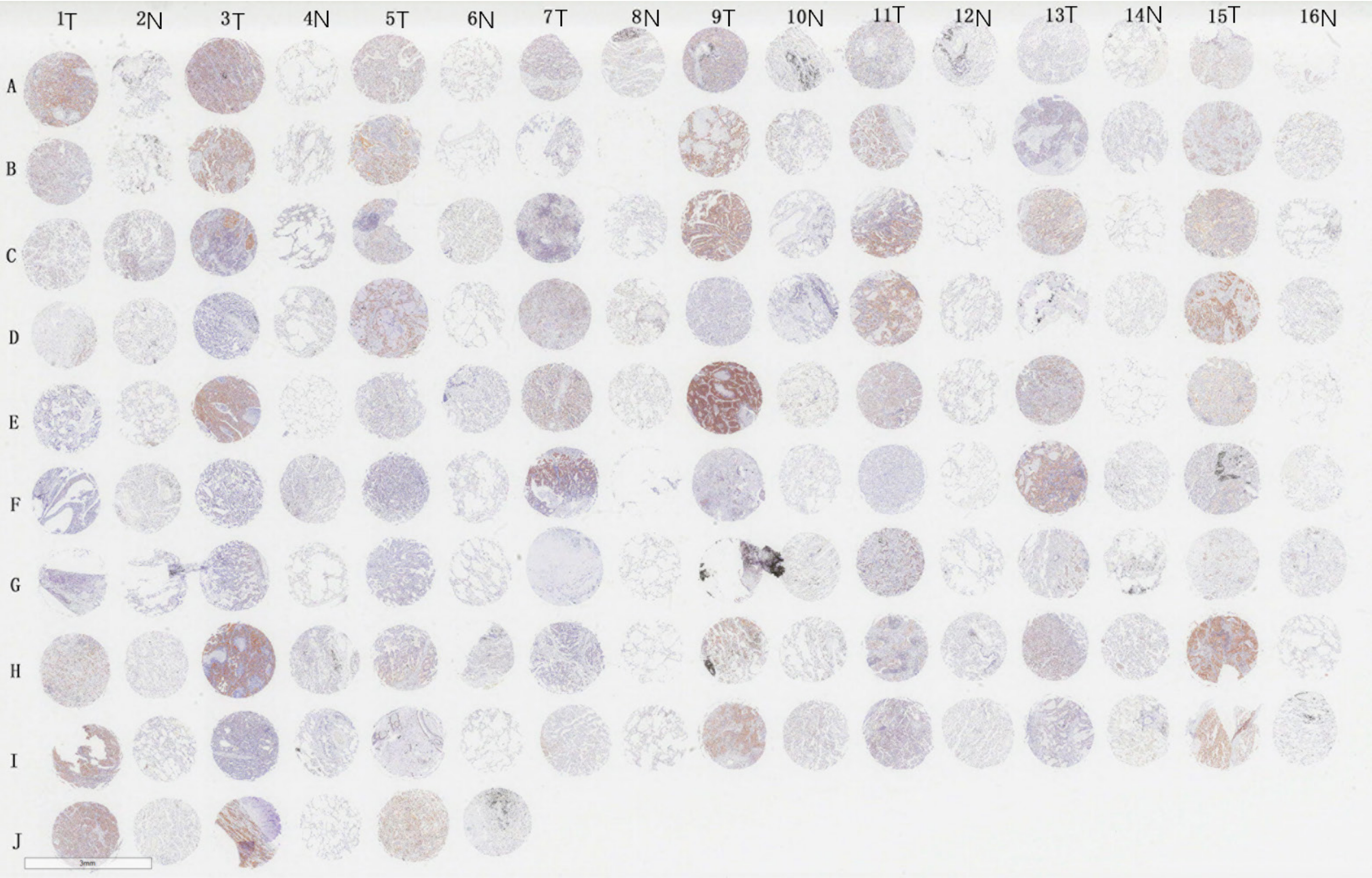

Supplementary Figure 1 Specificity and cellular localization of UCHL5 were determined by immunohistochemistry for the LUAD patients. A total of 75 of paired tumor and noncancerous tissues from the LUAD patients were stained with anti-UCHL5 antibody. Scale bar, 3mm. LUAD: lung adenocarcinoma.
